# Neuro-Intensive Treatment Targeting Intracranial Hypertension Improves Outcome in Severe Bacterial Meningitis: An Intervention-Control Study

**DOI:** 10.1371/journal.pone.0091976

**Published:** 2014-03-25

**Authors:** Martin Glimåker, Bibi Johansson, Halla Halldorsdottir, Michael Wanecek, Adrian Elmi-Terander, Per Hamid Ghatan, Lars Lindquist, Bo Michael Bellander

**Affiliations:** 1 Unit for Infectious Diseases, Department of Medicine Solna, Karolinska Institutet and Karolinska University Hospital, Stockholm, Sweden; 2 Unit for Infectious Diseases, Department of Medicine Huddinge, Karolinska Institutet and Karolinska University Hospital, Stockholm, Sweden; 3 Department of Anesthesiology, Karolinska University Hospital, Stockholm, Sweden; 4 Department of Clinical Neuroscience, Section for Neurosurgery, Karolinska University Hospital, Stockholm, Sweden; 5 Department of Clinical Neuroscience, Karolinska Institutet, Stockholm, Sweden; Glaxo Smith Kline, Denmark

## Abstract

**Objective:**

To evaluate the efficacy of early intracranial pressure (ICP)-targeted treatment, compared to standard intensive care, in adults with community acquired acute bacterial meningitis (ABM) and severely impaired consciousness.

**Design:**

A prospectively designed intervention-control comparison study of adult cases from September 2004 to January 2012.

**Patients:**

Included patients were confirmed ABM-cases, aged 16–75 years, with severely impaired mental status on admission. Fifty-two patients, given ICP-targeted treatment at the neuro-intensive care unit, and 53 control cases, treated with conventional intensive care, were included. All the patients received intensive care with mechanical ventilation, sedation, antibiotics and corticosteroids according to current guidelines. Additional ICP-treatment in the intervention group included cerebrospinal fluid drainage using external ventricular catheters (n = 48), osmotherapy (n = 21), hyperventilation (n = 13), external cooling (n = 9), gram-doses of methylprednisolone (n = 3) and deep barbiturate sedation (n = 2) aiming at ICP <20 mmHg and a cerebral perfusion pressure of >50 mmHg.

**Measurements:**

The primary endpoint was mortality at two months and secondary endpoint was Glasgow outcome score and hearing ability at follow-up at 2–6 months.

**Outcomes:**

The mortality was significantly lower in the intervention group compared to controls, 5/52 (10%) versus 16/53 (30%; relative risk reduction 68%; p<0.05). Furthermore, only 17 patients (32%) in the control group fully recovered compared to 28 (54%) in the intervention group (relative risk reduction 40%; p<0.05).

**Conclusions:**

Early neuro-intensive care using ICP-targeted therapy, mainly cerebrospinal fluid drainage, reduces mortality and improves the overall outcome in adult patients with ABM and severely impaired mental status on admission.

## Introduction

Acute bacterial meningitis (ABM) in adults is associated with a considerable risk of death and neurological deficits [1]–[6]. Impaired mental status, high age, co-morbidity, non-meningococcal etiology and fulminant disease are reported risk factors for poor outcome [2], [5], [7], [8]. Patients presenting a Glasgow coma scale (GCS) <9 on admission present a significantly higher mortality rate compared to patients presenting GCS >12 [2], [8]–[12]. ABM is associated with increased intracranial pressure (ICP) [13]–[17], which may cause a reduced cerebral blood flow and/or brain herniation [2], [18]–[21]. The standard of care for acute bacterial meningitis includes initiation of adequate antibiotics and corticosteroids in meningitis doses within one hour of admission, and if impaired mental status or other signs of increased ICP, intensive care with proper analgesia and assisted mechanical ventilation. If a severe septic syndrome is present the management follows the current routines for severe sepsis. Moderate hyperventilation, osmotherapy and ICP-targeted treatment can be considered in critical cases of ABM [7], [22]–[24], and is well established in patients suffering from traumatic brain injury (TBI) [25]. A neurocritical care approach using ICP-targeted treatment with favorable results has been reported in three relatively small and uncontrolled studies of ABM-patients presenting high ICP and severe impairment of consciousness [14]–[16], and promising results have recently been reported in a cohort study using lumbar drainage [23]. However promising, there is a lack of evidence supporting ICP-guided treatment in ABM [7], [22].

The objective of the present study was to test the hypothesis that ICP-targeted treatment, mainly cerebrospinal fluid (Csf) drainage through an external ventricular drainage (EVD), could improve the outcome in adults with community acquired ABM and severely impaired mental status on admission.

## Materials and Methods

A prospectively designed intervention-control comparison study was performed between September 2004 and January 2012. The primary endpoint was mortality at two months and the secondary endpoint was neurological and auditory deficits at 2–6 months follow-up.

### Ethics

The study was approved by the local ethic committee at Karolinska University Hospital including retrospective analyses of the Swedish National Quality Registry for ABM (SQRM) and patient records regarding the control group. Informed consent was obtained from relatives to patients included in the intervention group. Inclusion in the study had to be done directly on admission, when relatives often were absent. Consequently, written informed consent could often not be obtained in the intervention group. If not possible on admission, a verbal consent by telephone from relatives was obtained which was documented in the patient records. The control patients were informed that they were included in SQRM, and that this registry could be used for research. However, they were not informed about the retrospectively assembling of data in the present study. These procedures were approved by the ethics committee.

### Patient selection

Inclusion criteria were:

1. Age 16–75 years,
2. Confirmed ABM defined as a: positive Csf-culture and/or Csf-polymerase chain reaction (PCR), and/or b: positive blood culture with meningitis associated bacteria (*S pneumoniae*, *N meningitidis*, *H influenzae*, *L monocytogenes*) combined with Csf-findings consistent with ABM (increased cell-count and/or lactate concentration and/or decreased Csf/serum-glucose ratio) and/or clinical findings indicative of ABM (fever, headache and neck stiffness), and
3. Severely impaired mental status on admission defined as GCS ≤9, or GCS = 10 combined with lumbar spinal opening pressure >400 mmH_2_O, and/or reaction level scale (RLS) [26] ≥4, or RLS = 3 with localizing painful stimuli corresponding to GCS motor score of 5 [14], [26], [27].

#### Intervention group

**Figure 1:**
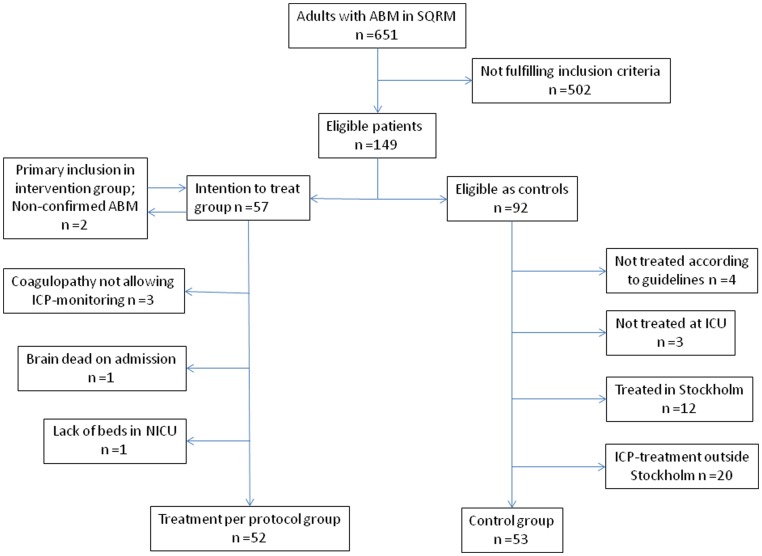
The algorithm for inclusion and exclusion of patients. Inclusion criteria were: 1) age 16–75 years, 2) severely impaired mental status on admission, and 3) confirmed acute bacterial meningitis (ABM). Patients were initially included in the intervention group based on clinical suspicion of ABM with or without cerebrospinal fluid analysis. SQRM = Swedish National Quality Registry for ABM. ICP = intracranial pressure. NICU = neuro-intensive care unit. ICU = intensive care unit.

Patients in the intervention group were recruited from seven hospitals in the Stockholm region, serving about 2.5 of Sweden's 9 million inhabitants. The neuro-intensive care unit (NICU) at the Karolinska University Hospital Stockholm was contacted for preliminary inclusion immediately after admission when identifying a patient with a strong suspicion of ABM based on clinical grounds, with or without Csf-analyses, and inclusion criteria 1 and 3 above. Definite inclusion was made after the microbiological analyses. Fifty-nine patients were preliminarily eligible for the intervention group. Two patients were excluded due to non-confirmed ABM diagnosis, and the remaining 57 cases constituted the intention to treat group (Figure 1). Five patients were excluded due to severe coagulopathy (n = 3), lack of beds at the NICU (n = 1), and brain death with computerized tomography (CT)-verified cerebral infarction and herniation on admission (n = 1). Thus, finally 52 patients were included in the per protocol analysis of the intervention group.

#### Control group

The controls were included retrospectively from clinical admission data registered prospectively in SQRM. From this registry, 651 consecutive patients were identified (Figure 1). This constitutes an estimated 75% of all adult cases with community acquired ABM in Sweden, according to data from The National Board of Health and Welfare, during the study period. Of 651 patients 149 (23%) fulfilled the above inclusion criteria. Of these, 57 were included in the intervention group and the remaining 92 cases were eligible as controls. To avoid a possible selection bias the following controls were excluded by independent observers; 1) patients not treated according to national guidelines including initial corticosteroids (dexamethasone or betamethasone) and adequate antibiotics (cefotaxime or ceftriaxone +/− ampicillin, or meropenem) in meningitis dosage (n = 4), and 2) patients not treated at an intensive care unit (ICU) with assisted ventilation and sedation (n = 3; one of these was judged desolate on admission). Moreover, 3) eligible patients from the Stockholm region, where treating physicians had missed to contact NICU for inclusion in the intervention group (n = 12), and 4) patients where ICP-targeted treatment in a NICU outside Stockholm was performed, independently of this study (n = 20), were not included in the final control group. The outcomes of these 20 cases and the 12 eligible patients from Stockholm are presented separately. The remaining 53 patients, admitted to one of 18 Regional or University hospitals outside Stockholm, constituted the final control group. ICP-targeted treatment in a NICU outside Stockholm was not withheld due to poor prognosis in any of these controls according to independent observers.

Inclusion or exclusion based on assessing consciousness was performed using GCS in all cases in the intervention group and in 14 controls, whereas 39 control patients were included according to RLS, as this is used in favor of GCS in many Swedish hospitals.

### Intensive care

All patients were treated in an ICU with adequate antibiotics and corticosteroids in ABM-doses. Standard intensive care management according to current recommendations regarding severe ABM [7] with mechanical ventilation, adequate sedation, and aiming at normal fluid and electrolyte homeostasis, mean arterial pressure ≥65 mmHg, SaO_2_ ≥95%, pCO_2_ 4.5–5.5 kPa, and blood-glucose 5–10 mmol/L, was administered to all controls and initially to the intervention group.

### ICP-targeted treatment in the intervention group

The decision to perform ICP-targeted treatment, in addition to standard intensive care, was made on admission in all intervention patients. Thirty-five patients were transported directly to the NICU whereas 17 cases, primarily admitted to one of six other hospitals in the Stockholm region, were treated at local ICUs during 1–36 hours, before being referred to the NICU. Following CT-scanning of the brain, an EVD-catheter for ICP-monitoring was established in 50 patients. In two of these the EVD did not function due to slit ventricles. In these two and in another two cases, where an EVD could not be applied (technical problems: n = 1, moderate coagulopathy: n = 1) a parenchymal ICP-monitor (Codman, Johnson and Johnson Nordic AB, Stockholm) was used. Patients were treated in a 30° sitting position. The temple was used as zero-point for mean arterial blood pressure and cerebral perfusion pressure. ICP was continuously registered in a computerized patient monitoring system, ICU-pilot (μ-dialysis AB, Solna, Sweden), and the treatment goals were ICP <20 mmHg and cerebral perfusion pressure >50 mmHg. Significant intracranial hypertension was defined as one or more episodes of ICP, continuously exceeding 20 mmHg for more than 5 minutes.

**Figure 2:**
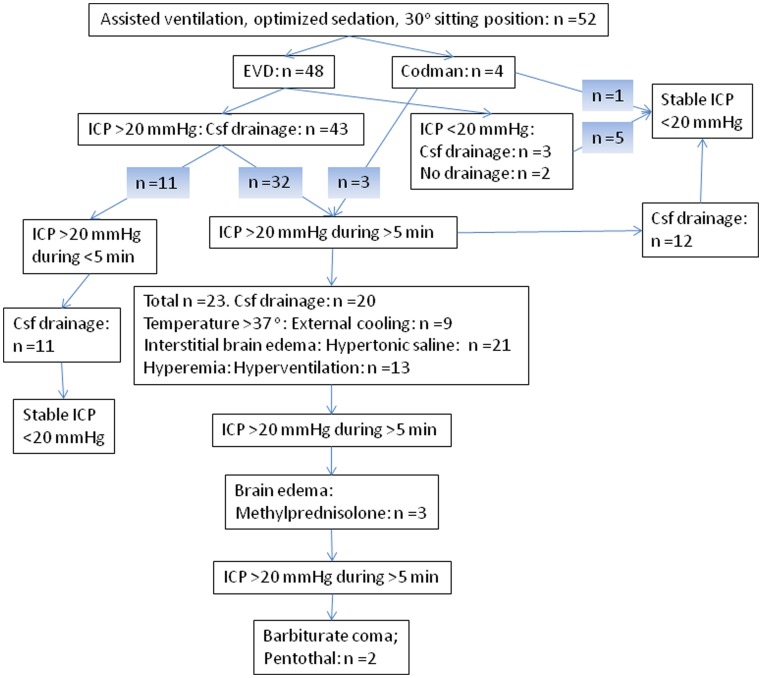
The algorithm for treatment of increased intracranial pressure (ICP) in the neuro-intensive care unit (n = 52). An external ventricular drainage (EVD) was established at operation in 50 patients with initial cerebrospinal fluid (Csf) drainage in 48 of these. A parenchymal ICP-monitor (Codman) was inserted in four cases. Bolus doses of Mannitol were administered in five cases prior to neuro-intervention. The shaded numbers represent different patients.

As illustrated in figure 2, CSF-drainage through the EVD was the main treatment of increased ICP. The upper ICP limit triggering drainage was set at 20 mmHg. Additional ICP treatment was applied at the discretion of the treating physicians, guided by clinical, radiological and physiological findings. If signs of interstitial brain edema were observed on the CT-scan, bolus doses of hypertonic saline were given as osmotherapy, allowing S-Na levels of not higher than 160 mmol/L. Intracranial hypertension due to hyperaemia, detected and guided by trans-cranial doppler and/or jugular bulb monitoring, was treated with moderate hyperventilation aiming at a pCO_2_ of 4.0–4.5 kPa. In cases with paracetamol resistant hyperthermia, an external cooling blanket (Thermo-wrap) was used, aiming at normothermia. If intracranial hypertension and brain edema persisted, despite previous corticosteroid treatment and above-mentioned ICP-decreasing interventions, 1 g of i.v. methylprednisolone was given. Last resort ICP treatment was a pentothal infusion, inducing a barbituate coma to lower cerebral metabolism, monitored with microdialysis and/or jugular bulb analyses.

### Clinical controls and follow-up

Clinical, etiological and laboratory findings, mental status (GCS and/or RLS) on admission, and the duration from admission to start of antibiotic and corticosteroid treatment were prospectively registered in the SQRM (Table 1). A CT-scan of the brain was performed in all patients in the intervention group and, based on clinical grounds, in 50 of the 53 cases in the control group. EEG was performed in selected cases to exclude non-convulsive seizures. A follow-up at 2–6 months after discharge included an evaluation of neurological deficits, Glasgow outcome score (GOS) and hearing impairment in all survivors according to the SQRM protocol. Audiometry was performed in selected cases.

**Table 1:** The main characteristics regarding demographic, etiological, clinical, and laboratory data, not identifying any significant difference between the intervention and control group, including comparison of the reaction level scale (RLS) values that were converted from Glasgow coma score (GCS) in the intervention group [27], [43].

	Intervention per protocol group	Control group	P-value^e^
	n (%)	n (%)	
	Total 52 (100)	Total 53 (100)	
**Females/males**	28/24	24/29	0.44
**Age (years):** Median	55	58	0.74
Range	16–74	18–74	
**Immunocompromised state:**	20^a^ (38)	[n = 52] 15^b^ (29)	0.41
**Duration of CNS symptoms before primary admission:**	[n = 51]	[n = 48]	
>24 hours	6 (12)	5 (10)	1.00
12–24 hours	15 (29)	17 (35)	0.67
<12 hours	30 (59)	26 (54)	0.69
**Seizures on admission:**	13 (25)	[n = 51] 11 (22)	0.82
**Mental status on admission:**			
GCS ≤7/RLS ≥5	27 (52)	21 (40)	0.24
GCS ≤4/RLS = 8	5 (10)	3 (6)	0.49
**Lumbar puncture, Csf findings on admission:**	47 (90)	44 (83)	0.39
Spinal opening pressure >400 mmH_2_O	[n = 21] 20 (95)	[n = 10] 9 (90)	1.00
Leukocyte count (×10^6^/L)	[n = 47]	[n = 44]	
Median	2400	2550	1.00
Range	7–34100	10–30500	
<1000	16 (34)	16 (36)	0.83
<100	4 (9)	5 (11)	0.73
Lactate (mmol/L)	[n = 43]	[n = 33]	
Median	12.4	14.6	0.92
Range	6.0–30.8	5.0–33.0	
**Blood platelets (×10^9^/L) on admission:**		[n = 51]	
<100	10 (19)	6 (12)	0.42
<50	0	2 (4)	0.24
**Time from primary admission to antibiotic treatment:**			
Range (hours)	0–14	0–12	
<2 hours	37 (71)	29 (56)	0.71
<1 hour	17 (33)	19 (37)	0.84
**Etiology:**			
*S pneumoniae*	41 (79)	36 (68)	0.27
*N meningitidis*	8 (15)	8 (15)	1.00
Other bacteria	3^c^ (6)	9^d^ (17)	0.12

LP = lumbar puncture. Csf = cerebrospinal fluid.

When data was missing the numbers of patients with available data are shown [in brackets].

a Malignancy/immunosuppresion: n = 10, Alcoholism: n = 5, Diabetes: n = 3, Splenectomized: n = 1, CSF-leakage: n = 1,

b Malignancy/immunosuppresion: n = 5, Alcoholism: n = 3, Diabetes: n = 3, Splenectomized: n = 2, CSF leakage: n = 2.

c *S aureus*: n = 2, *L monocytogenes*: n = 1.

d *S aureus*: n = 4, *H influenzae*: n = 2, *L monocytogenes*: n = 1, *S pyogenes* geoup C: n = 1, *E cloacae*: n = 1.

e Two tailed Fisher's exact test for proportions and Student T-test for normally distributed values.

### Statistical analyses

A power test was performed, based on available data [2], [8]–[10], [12], [14]–[16] suggesting a mortality of 40% with standard ICU treatment, and hypothesizing a 20% mortality when employing ICP-targeted treatment of ABM patients with severely impaired mental status at the NICU. Consequently, at least 50 patients are required in each group to achieve a probability of 80% to detect a 20% difference in mortality with a 95% CI. A two-tailed Fisher's exact test was used when comparing groups regarding outcomes, demographic, etiological and clinical data. The Student T-test was used for the comparison of laboratory values. Multivariate analyses were performed to adjust for possible differences in case mixes of the control and intervention group. (Table 1).

## Results

The main characteristics regarding demographic, etiological, clinical, and laboratory data are given in Table 1, not identifying any significant differences between the intervention and control group. A cerebral CT-scan on admission showed one or more of the following; tight sulci, edema, compressed cisterns or hydrocephalus, in 22/52 (42%) patients in the intervention group. However, CT-findings were lacking in the controls as this data was not recorded in the SQRM.

### ICP management in the intervention group

The median time from primary admission to insertion of an EVD or a parenchymal ICP-monitor was 8 h∶26 min (range 1 h∶27 min–96 h) and the median duration of EVD management was 5 days (range 1.5–16 days).

Five patients were treated with bolus doses of Mannitol upon admission, due to slit ventricles as seen on the CT-scan (n = 3), or a spinal opening pressure of >500 mmH_2_O (n = 2). An EVD was applied in 48 cases, and a parenchymal ICP-monitor was inserted in 4 cases (slit ventricles; n = 2, technical problems; n = 1, coagulopathy; n = 1). When the EVD was applied, Csf was momentarily drained in the operating room in 48 cases, and in 38 (79%) of these cases, the neurosurgeon noted markedly elevated ICP during this procedure.

**Figure 3:**
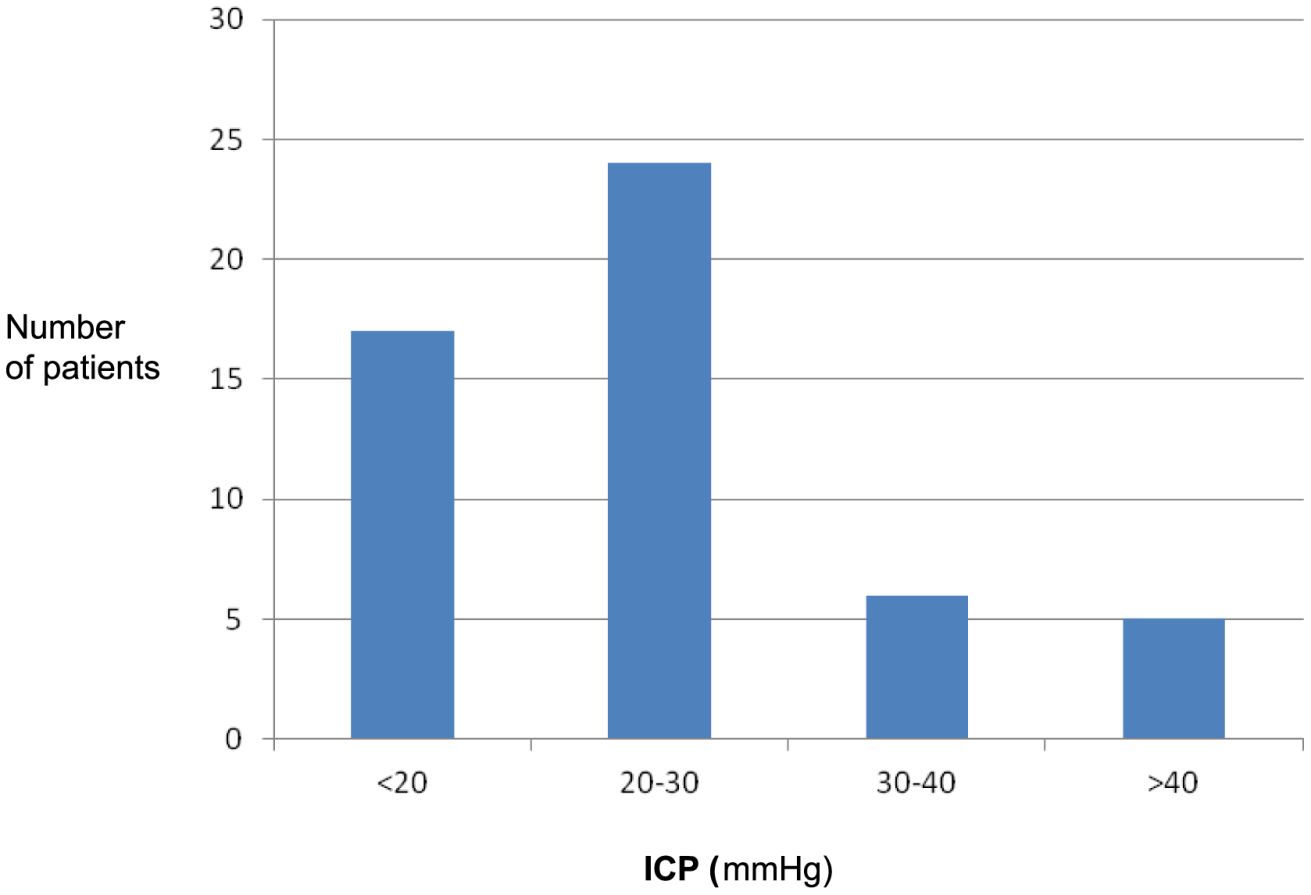
The highest levels of intracranial pressure (ICP), as observed continuously during episodes of more than 5 min, in the neuro-intensive care unit after initial cerebrospinal fluid drainage at operation; n = 52 (external ventricular drainage; n = 48, parenchymal ICP-monitor; n = 4).

In the NICU, 35 of 52 patients (67%) presented significant intracranial hypertension (ICP >20 mmHg during >5 min; Figure 3). A parenchymal ICP-monitor but not an EVD was established in three of these 35 cases. Of the remaining 17 patients, 11 exhibited ICP >20 mmHg, but for shorter episodes than 5 min. In these cases, Csf was also drained through the EVD to maintain an ICP <20 mmHg. In three additional cases, Csf was drained despite ICP <20 mmHg (Figure 2). Thus, Csf-drainage was performed in altogether 46 (88%) of the 52 cases. Four of the remaining six patients had parenchymal probes instead of EVD.

In aggregate, increased ICP at surgery and/or significant intracranial hypertension during the NICU-care was observed in altogether 43 (83%) of the 52 patients (Table 2). The peak levels of ICP occurred within the first 48 hours of NICU-care in all patients, except in one patient who exhibited the highest level on day three. Altogether, 49 (94%) of the intervened patients were administered specific ICP-decreasing treatment in the NICU (Figure 2, Table 3).

**Table 2:** The intracranial pressure (ICP; mmHg) as noted at operation (external ventricular drainage: n = 48; parenchymal ICP-monitor: n = 4), and the highest levels of ICP, noted continuously during episodes >5 min, at the neuro-intensive care unit (NICU), related to the outcome at follow-up after 2–6 months in the intervention per protocol group.

ICP at operation	Normal	Elevated	Elevated or not analysed			
ICP in NICU	<20	<20	20–30	30–40	>40	
	n = 9	n = 8	n = 24	n = 6	n = 5	Total n = 52
**Outcome:**						
**Recovery; GOS 5 and normal hearing**	5	4	14	4	1	28
**GOS 5 and impaired hearing**	0	0	3	0	1	4
**Neurological deficit; GOS 2–4** *	4^a^	4^b^	5^c^	2^d^	0	15
**Death; GOS 1**	0	0	2	0	3	5

GOS = Glasgow outcome score.

*These patients ended up as follows;

a) Two with GOS 3, two with GOS 4.

b) Four with GOS 4.

c) One with GOS 3, four with GOS 4.

d) Two with GOS 4.

**Table 3:** Intracranial pressure (ICP)-targeting therapy used in the intervention group, (n = 52, external ventricular drainage; n = 48, parenchymal ICP-monitor; n = 4).

	n	%
Mannitol prior to intervention	5	10
Drainage of Csf at operation	48	92
Drainage of Csf at NICU	46	88
Hyperosmolar therapy	21	40
Hyperventilation	13	25
External cooling	9	17
Methylprednisolone	3	6
Barbiturate coma	2	4
No ICP-treatment in addition to deep sedation and mechanical ventilation in 30° sitting position	3	6

Csf = cerebrospinal fluid; NICU = neuro-intensive care unit.

### Outcome

Mortality was significantly decreased in the per protocol intervention group (5/52 = 10%) compared to the controls (16/53 = 30%; relative risk reduction 68%; p<0.05; Table 4). Full recovery, defined as GOS 5 with preserved normal hearing, was significantly more frequent in the per protocol group; 28 patients (54%) versus 17 patients (32%) in the control group. Thus, the relative risk reduction for unfavorable outcome was 40% (p<0.05).

**Table 4:** Outcomes at follow-up after 2–6 months in the intervention group and in the controls.

	Intervention group		Eligible as control patients		
Outcome	Intention to treat group	Per protocol group	Control group^a^	Cases from Stockholm missed for intervention	NICU-treated cases outside Stockholm
	n = 57 (%)	n = 52 (%)	n = 53 (%)	n = 12 (%)	n = 20 (%)
**Recovery; GOS 5 and normal hearing**	30 (53)*	28 (54)*	17 (32)*	4 (33)	8 (40)
**GOS 5 and impaired hearing**	4 (7)	4 (8)	6 (11)	1 (8)	1 (5)
**GOS 2–4 +/− impaired hearing**	16 (28)	15 (29)	14 (26)	4 (33)	6 (30)
**Death; GOS 1**	7 (12)*	5 (10)*	16 (30)*	3 (25)	5 (25)

NICU = neuro-intensive care unit. GOS = Glasgow outcome score.

*p<0.05 with two-tailed Fisher's exact test when comparing the intention to treat group, and the per protocol group, with the control group.

a Patients included as final controls according to inclusion and exclusion criteria.

Three intervened patients suffered persisting severe neurological deficits, with outcomes of GOS 3, related to complications of ABM; sinus/intracranial venous thrombosis; n = 1, cerebral infarction; n = 1, and ventriculitis and hydrocephalus; n = 1. The remaining 44 survivors reached GOS 4 (n = 12) or 5 (n = 32; Table 2). Of the 28 intervened patients that fully recovered, including normal hearing, 19 (68%) had significant intracranial hypertension in the NICU and a further four had markedly elevated ICP noted only at surgery (Table 2). Significantly more episodes of ICP >20 mmHg lasting >5 min were observed among patients that succumbed, versus survivors (p<0.01). Slit ventricles, not allowing drainage through the EVD, were found in three of the five fatal cases.

All deaths occurred within one month after admission. Three of the five fatal cases in the treatment group died within a week, as did 11/16 fatalities among the controls. Two of the five fatalities in the intervention group, and 6 of the 16 controls that died, were immunocompromised (Table 1). *S pneumoniae* was the etiological agent in all fatal cases of the intervention group, and in 9 of 16 succumbed controls. The causes of mortality were cerebral herniation and/or ischaemia, verified by CT-scan and/or autopsy, in 4/5 (80%) intervened patients and in 13/16 (81%) controls. In the remaining cases, death was caused by septic shock and/or multi-organ failure.

Lumbar puncture (LP) was avoided due to contraindication in two (suspected herniation in both) of the five fatal cases in the intervention group, and in four (suspected herniation: n = 2, coagulopathy: n = 2) of the 16 controls that died. Thus, the mortality rate among spinal tapped patients (Table 1) was 3/47 (6%) and 12/44 (27%), in the intervention and control groups, respectively. No patient died within 24 hours after LP and, of the intervened patients with persisting deficit, a significant clinical deterioration shortly after LP was not recorded in any case.

## Discussion

Acute bacterial meningitis (ABM) in adults still remains a challenge for the clinician, and in comatose ABM-patients mortality rates of up to 62% have been reported [2], [9], [10]. ICP-targeting treatment of severe ABM has been reported with promising results [14]–[16], [23] but is not routinely used. [4], [22].

The present report is, to our knowledge, the first controlled study of neuro-intensive care and ICP-guided therapy using EVD in adults with ABM and severely impaired consciousness. The mortality was significantly decreased from 30% in the controls to 10% in the treatment group, and the incidence of full recovery was increased from 46% to 68% in the groups, respectively. Significant initial intracranial hypertension was observed in two thirds of the 28 patients that fully recovered, indicating that ICP-guided therapy may result in a satisfactory outcome, even in cases with severe ICP elevation. However, in concordance with earlier findings, extreme ICP levels were observed among the fatal cases [15]–[17], [19], [20], [28]. The peak ICP occurred early during the course of the disease, as shown earlier [14]–[16].

In line with previous experience, cerebral herniation and/or infarction dominated as cause of mortality in both groups [2], [13]–[16], [18], and a very aggressive course of ABM with fatal outcome was observed in five intervened cases [20], [29], [30]. A few case reports suggest that a decompressive craniectomy may be considered early in the course of disease in such cases [31]–[33].

The pathophysiological mechanisms resulting in increased ICP in ABM are multifactorial. The release of bacterial components in the subarachnoid space leads to an inflammatory response that contributes to increased permeability of the blood-brain barrier causing cerebral extracellular edema, impaired Csf-absorption with increased Csf-volume, a cytotoxic intracellular brain edema, and increased cerebral blood flow (hyperaemia); all adding to elevated ICP [34], [35].

Our study suggests that increased ICP is a major contributor to mortality and morbidity in ABM, and that Csf-drainage significantly affects recovery. Both the initial Csf-drainage during EVD insertion and further drainage in the NICU have likely contributed to favorable outcome by decreasing ICP, consistent with earlier studies [14], [15]. Additional ways of achieving ICP control may also be effective. Osmotic therapy has been suggested in earlier studies of TBI [36] and ABM [16], and hypertonic saline was administered to 40% of the patients in the present study. The use of Mannitol for osmotic treatment of elevated ICP is controversial because of a potential risk of rebound increase in ICP [37]. Hyperventilation was performed in 13 cases in our study indicating that this strategy may be valuable, especially during hyperaemia, as suggested earlier [14].

All these treatments require some modality of ICP measurement. This was achieved by an EVD in the vast majority of patients in our study. However, an EVD could not be applied in three cases due to severe coagulopathy showing the limited utility of neuro-intervention in cases with severely disturbed hemostasis, which may occur especially in meningococcal disease. In cases with moderate coagulopathy a parenchymal ICP-monitor may be an alternative option [16]. Favorable outcome has recently been reported in 11 adults with severe ABM by means of lumbar Csf-drainage, a method associated with decreased risk of complications but less appropriate ICP-monitoring compared with an EVD or a parenchymal ICP-monitor [23].

Early and adequate management of ABM is vital. Correct treatment is, in clinical practice, often delayed until LP has been performed [5], [38]–[40]. In this study, rapid diagnosis was achieved in the vast majority of patients by comparably early LP. This contributed to early antibiotic and corticosteroid treatment in both study groups. Importantly, significant LP associated complications were not noticed in the present study. Additionally in our study, the preliminary inclusion criteria we applied have likely contributed to early neuro-intervention (median 8.5h from admission), compared to NICU-treated patients outside the Stockholm region (according to SQRM) with a mortality of 25% in this study, and those NICU treated cases in the earlier studies with mortality rates of about 30% [14], [16]. A high diagnostic accuracy (97%) was seen with our preliminary criteria of clinical signs with or without Csf-analysis. Furthermore, measurement of lumbar pressure was instrumental to inclusion of two cases. Although not observed in our study, EVD-insertion may cause side effects [41], [42] which further emphasizes the significance of rapid and reliable ABM-diagnosis. In aggregate, the timing of neuro-intervention and the inclusion criteria, enabling early identification of patients at risk, may be important to achieve favorable outcome.

Some limitations were identified of the present study: The main limitation is the lack of randomization. Controls were identified retrospectively, but they had been prospectively included in the SQRM database comprising inclusion and exclusion criteria, as well as outcome information. The majority of controls were included according to RLS, whereas all patients in the intervention group were included according to GCS. This discrepancy could cause a bias, but the GCS is reliably converted to RLS [27], [43]. A study protocol was lacking for controls and care differs at various centers, as it relates to institutional protocols as well as the availability of subspecialists. Therefore, extrapolating outcomes in different settings may be questionable. However, the control group received standard intensive care according to current guidelines, and specialists in infectious diseases were involved in all cases, and other specialists were consulted when appropriate. Furthermore, outcome among controls was favourable in comparison with recent studies of ABM with severely impaired consciousness [8], [12].

Great attention was paid to include all consecutive patients nationwide outside Stockholm as controls to avoid selection bias. The initial intention was to match the controls for age, etiology and mental status, but all eligible patients that fulfilled the inclusion criteria had to be included due to limited number of cases. Twelve Stockholm-cases were missed for neuro-intervention, and 20 patients were excluded from the final control group because of NICU-treatment outside Stockholm. Thus, a possible selection bias cannot be ruled out. However, ICP-targeted treatment was not withheld due to poor prognosis in any of these controls. Moreover, if the Stockholm-cases missed for intervention, and/or if the NICU-treated patients outside Stockholm were included in the control group, the significant difference in outcome between the treatment group and controls did not change.

Finally, 23% of the patients, registered in SQRM, were eligible for the present study, i.e. presented with severely impaired mental status on admission, and about half of the patients, included in the study, were comatose (GCS ≤7 and/or RLS ≥5). These proportions of cases with severely impaired mental status and coma, respectively, are consistent with earlier findings on admission of large materials with adult ABM [5], [8], [40], [44], indicating that the study population was representative. Non-meningococcal etiology is shown to be a risk factor for unfavorable outcome [5]. In the present study, equal proportions (15%) of meningococcal etiologies were found in the two groups, but the percentage of pneumococcal ABM was higher in the intervention group (79% versus 68%). In contrast, more cases with other bacteria were found in the control group. However, it is unlikely that this difference in etiology was a confounder, and if so would favor the control group, as mortality is higher among pneumococcal ABM.

In conclusion, although the limitations should be considered, the two study groups were clinically comparable, and all the controls were treated adequately with antibiotics, corticosteroids and standard intensive care according to current guidelines. The large decrease in mortality and increase of favorable outcome, support the benefit of neuro-intervention in selected ABM cases. Despite lacking a randomized clinical trial, we suggest that early ICP-specific treatment in NICU with Csf-drainage should be considered on admission in patients with ABM and severely impaired mental status. A randomized study on ICP-targeted treatment versus traditional standard of care in ABM would be desirable. However, the unequivocal results of this study make Csf-drainage as a basic treatment modality difficult to exclude. Therefore, a study comparing Csf-drainage by EVD with lumbar drainage would be more appropriate.
